# Serum-Free and Xenobiotic-Free Preservation of Cultured Human Limbal Epithelial Cells

**DOI:** 10.1371/journal.pone.0118517

**Published:** 2015-03-03

**Authors:** Oeygunn Utheim, Rakibul Islam, Torstein Lyberg, Borghild Roald, Jon Roger Eidet, Maria Fideliz de la Paz, Darlene A. Dartt, Sten Raeder, Tor Paaske Utheim

**Affiliations:** 1 Department of Medical Biochemistry, Oslo University Hospital, Oslo, Norway; 2 Department of Ophthalmology, Oslo University Hospital, Oslo, Norway; 3 Department of Oral Biology, Faculty of Dentistry, University of Oslo, Oslo, Norway; 4 Department of Pathology, Oslo University Hospital, Oslo, Norway; 5 Institut Universitari Barraquer, Universitat Autonoma de Barcelona, Barcelona, Spain; 6 Schepens Eye Research Institute / Massachusetts Eye and Ear Infirmary, Department of Ophthalmology, Harvard Medical School, Boston, MA, United States of America; 7 The Norwegian Dry Eye Clinic, Oslo/Tromsoe, Norway; University of Newcastle upon Tyne, UNITED KINGDOM

## Abstract

**Aim/Purpose of the Study:**

To develop a one-week storage method, without serum and xenobiotics, that would maintain cell viability, morphology, and phenotype of cultured human limbal epithelial sheets.

**Materials and Methods:**

Human limbal explants were cultured on intact human amniotic membranes for two weeks. The sheets were stored in a hermetically sealed container at 23°C in either a serum-free medium with selected animal serum-derived compounds (Quantum 286) or a xenobiotic-free medium (Minimal Essential Medium) for 4 and 7 days. Stored and non-stored cultures were analyzed for cell viability, amniotic membrane and epithelial sheet thickness, and a panel of immunohistochemical markers for immature cells (ΔNp63α, p63, Bmi-1, C/EBP∂, ABCG2 and K19), differentiated cells (K3 and Cx43), proliferation (PCNA), and apoptosis (Caspase-3).

**Results:**

The cell viability of the cultures was 98 ± 1% and remained high after storage. Mean central thickness of non-stored limbal epithelial sheets was 23 ± 3 μm, and no substantial loss of cells was observed after storage. The non-stored epithelial sheets expressed a predominantly immature phenotype with ΔNp63α positivity of more than 3% in 9 of 13 cultures. After storage, the expression of ABCG2 and C/EBP∂ was reduced for the 7 day Quantum 286-storage group; (P = 0.04), and Bmi-1 was reduced after 4 day Quantum 286-storage; (P = 0.02). No other markers varied significantly. The expression of differentiation markers was unrelated to the thickness of the epithelia and amniotic membrane, apart from ABCG2, which correlated negatively with thickness of limbal epithelia (R = -0.69, P = 0.01) and ΔNp63α, which correlated negatively with amniotic membrane thickness (R = -0.59, P = 0.03).

**Conclusion:**

Limbal epithelial cells cultured from explants on amniotic membrane can be stored at 23°C in both serum-free and xenobiotic-free media, with sustained cell viability, ultrastructure, and ΔNp63α-positivity after both 4 and 7 days.

## Introduction

The cornea transmits light to the retina to enable vision. The outermost layer of the cornea, the epithelium, is renewed by stem cells located in the transitional zone between the cornea and the conjunctiva, called the limbal region [1,2]. Limbal stem cells can be damaged by a number of factors including chemical burns, autoimmune diseases, and infections such as trachoma. These issues may result in limbal stem cell deficiency (LSCD), a condition that can lead to both severe pain and blindness.

In 1997, Pellegrini *et al*. showed that ex vivo cultured human limbal epithelial cells (HLEC) can reconstruct the ocular surface following transplantation to patients with LSCD [3]. Since this pioneering work, several protocols for cultivating HLEC have emerged [4]; however, two major strategies are currently in use. The harvested epithelial cells can either be cultured from a cell suspension, in which single HLEC are released from the limbal tissue after enzymatic treatment [5], or cultured using the explant method, in which the limbal explant is placed in the center of a substrate in a culture dish, allowing cells to divide and migrate out from the explant [6].

Previous studies from our research group have shown that cultured HLEC sheets can be stored in a closed container for 7 days at 23°C in bovine serum-containing storage medium [7,8]. Storage technology offers several advantages, including flexibility for both the patient and the surgeon in scheduling of surgery [9], sufficient time for sterility testing and other quality assessments [7], and time to transport cultured tissue e.g. from the laboratory to the operating theatre.

In the present study, the main aim was to develop a xenobiotic-free storage method of cultured HLEC. A protocol free of fetal bovine serum (FBS) and other animal-derived components [10] is clearly preferred for several reasons. First, the use of serum carries a small but potentially lethal risk of transmitting prions, causing transmissible spongiform encephalopathy [11–13]. Second, serum and animal-derived components can transfer microorganisms; even if Good Manufacturing Practice facilities are implemented, low-level contamination may be missed [11,14,15]. Third, recipients of animal-borne components may experience severe immunologic reactions and even give rise to new viral epidemics [16–18]. The U.S. Food and Drug Administration (FDA) have issued strict guidelines against the use of xenogeneic cells during production of tissue, applying regulations on the same level as xenobiotic-transplantations [13]. Meeting FDA requirements is therefore difficult with the use of xenobiotics [19]. Hence, we aimed to investigate whether cultured HLEC could be stored under either serum-free or, most preferably, xenobiotic-free conditions for up to 1 week, while maintaining the morphology, cell viability, and phenotype.

We also determined the expression of the putative keratinocyte stem cell marker p63 [20] in the cultured HLEC sheets. In 2010, Rama *et al*. presented a study where 112 LSCD-patients were treated with cultures from autologous limbal cells using cell suspension. Interestingly, a successful post-operative outcome was not associated with the total number of clonogenic cells, but it correlated with the percentage of cells staining brightly for the transcription factor p63 [20] by immunofluorescence microscopy. The grafts that contained more than 3% p63-bright cells after culture were successful in 78% of the eyes, whereas transplants that contained smaller percentages of these cells were successful in only 11% of the eyes [21]. Cells expressing p63-bright cells were verified by clonal analysis to represent holoclone-forming cells (i.e., indicative of stem cells) [21–23]. Hence, the percentage of p63-bright cells in the graft detected by immunocytochemistry prior to surgery can serve as a key predictor for clinical outcome, without the need for additional clonal analysis [23].

P63 consists of six different isotypes of which only the ΔNα isotype is holoclone-associated [24]; therefore, we obtained a specific marker for ΔNp63α. In the present study, we examined whether ΔNp63α positivity of more than 3% per culture could be achieved with the explant cultivation technique as compared to cells in suspension, and whether or not the expression of ΔNp63α was sustained after storage.

We found that the cultured HLEC sheets maintained their morphology, cell viability, and phenotype after storage in both serum-free and xenobiotic-free media. In addition, 9 of 13 of the explant cultures achieved a ΔNp63α positivity of more than 3%, associated with a successful post-surgical outcome [21]. The latter suggests that not all HLECs cultured from explants contain an adequate number of stem cells. Storage technology would give time to detect unsuitable cultures that could be excluded prior to surgery [21]. Thus, our findings that the cultured HLEC sheets can be stored under xenobiotic free-conditions for 4 and 7 days opens the opportunity for quality testing of the cultured grafts prior to transplantation. Hence, our storage method could increase the ratio of successful limbal stem cell transplantations.

## Materials and Methods

### Experiment Design

Limbal explants from cadaveric donors were cultured on cryopreserved, intact human amniotic membranes (HAM) for three consecutive experiments. In the first and second experiment, explants from a total of five donors were distributed evenly between five groups (storage in Quantum 286 or MEM with L-Glutamine for 4 or 7 days, and a control group of non-stored cultures). Effort was also taken to distribute explants from the superior region of the limbal rings between the five groups, but not if this conflicted with an even distribution of donors. Cell viability, expression of immunohistochemical markers, and epithelial morphology for the 4 different storage conditions were compared with the non-stored control.

In addition, the data from the non-stored groups of the storage experiments were combined with data from a third experiment with HLEC sheets cultured with the same protocol, resulting in a group of 1–2 cultures per donor from a total of 10 donors. This data was used to explore variations in thickness of HAM and epithelial outgrowth between cultured HLEC sheets and to calculate possible correlations between thickness and expression of various immunohistochemical markers.

### Ethics Statement

The research was conducted in accordance with the Declaration of Helsinki. Written consent from either the donor or the next of kin was obtained by personnel at the Eye Bank of Barraquer Ophthalmology Centre for the use of limbal donor tissue for research purposes. The limbal donor tissue was shipped from Barcelona to Boston, U.S. where the first experiment was performed and from Barcelona to Oslo, Norway where the second and third experiments were performed. The appropriate authorities approved all the transfers. HAMs were donated after informed, written consent from healthy women who had undergone elective caesarian section at Oslo University Hospital, Norway. The Norwegian Regional Committee for Medical and Health Research Ethics approved the collection and banking of HAM and the use of ocular tissue.

### Culture and Preservation of the Epithelial Cells

**Fig 1:**
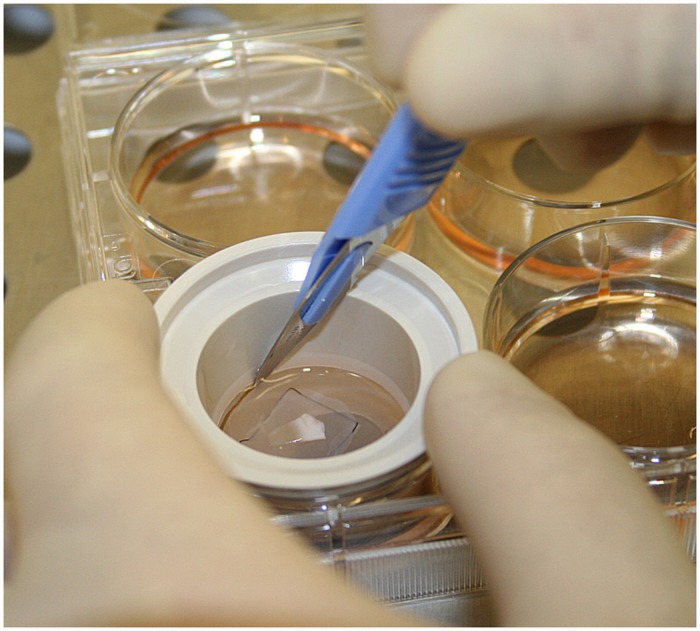
Preparation of Human Limbal Epithelial Cell Sheets. Photo showing preparation of a Human Limbal Epithelial Cell Sheet after 14- day culture prior to the transfer into storage containers. The polyester membrane insert is about to be cut out with a surgical blade, before being transferred to a storage container. In the center of the insert, a triangular shaped human limbal explant can be seen. The leading edge of the continuous epithelial sheet growing out from the explant is seen as a gray line outside the black suture in the picture.

Human amniotic membranes cryopreserved (not freeze dried) as previously reported [25] were thawed and attached to the polyester membrane of Netwell culture plate inserts (Corning, New York, USA) using 6–0 non-absorbable sutures. Limbal explants from cadaveric donors were prepared and cultured onto amniotic membranes as previously described by Meller *et al* [26] (Fig. 1). In short, limbal explants exposed to dispase (Roche Diagnostics, Basel, Switzerland) were incubated with the epithelial side facing the intact amniotic membrane at 37°C with 5% CO_2_ in a medium consisting of 4-(2-hydroxyethyl)-1-piperazineethanesulfonic acid (HEPES)-buffered Dulbecco’s modified Eagle’s medium containing sodium-bicarbonate and Ham’s F12 (Sigma-Aldrich, St Louis, Missouri, USA). The medium was supplemented with 5% fetal bovine serum, 0.5% dimethyl sulphoxide, 2 ng/mL human epidermal growth factor, 5 μg/mL insulin, 5 μg/mL transferrin, 5 ng/mL selenium, 3 ng/mL hydrocortisone, 30 ng/mL cholera toxin (Biomol, Exeter, UK), 50 μg/mL gentamycin, and 1.25 μg/mL amphotericin B [27] (Sigma-Aldrich). The medium was changed every 3^rd^ day. After 14 days of incubation, 17 cultures were analyzed directly, while the remaining 40 culture inserts were transferred from the plates containing culture media (Fig. 1) to radiation sterilized 90 mL Plastiques Gosselin polypropylene storage containers (Corning Life Sciences, Lowell, Massachusetts, USA) filled with 25 mL of storage medium. The cultures were subjected to storage in one of the two following media: 1) Minimal Essential Medium (MEM) with L-glutamine (Invitrogen, Carlsbad, USA), added 0.025 M HEPES, 0.024M sodium bicarbonate and 50 μg/mL gentamycin (hereafter referred to as MEM); or 2) Quantum 286 (PAA Laboratories GmbH, Pasching, Austria) added 50 μg/mL gentamycin. The containers were closed with a hinged cap with septum, placed in a wine cooler with a fixed temperature of 23°C, and left untouched for 4 or 7 days.

### Cell Viability Analysis

Viability staining was performed using a calcein-acetoxymethyl ester (CAM)/ethidium homodimer 1 (EH-1) (Invitrogen) assay [28] with some modifications. In brief, HLEC cultures prior to storage (n = 10), after 4 days of storage (n = 19), and after 7 days of storage (n = 14) were incubated in phosphate-buffered saline (PBS) containing 2 mM CAM and 2 mM EH-1 (23°C for 45 min, protected from light) and washed with PBS. Epithelial discs from the outgrowth zone of the cultures were trephined using a 6 mm Kai biopsy punch (Kai Industries, Gifu, Japan) and mounted on cover-slipped glass slides. Fluorescent images of the basal layer were recorded using an Axiovert 100 LSM 510 laser scanning confocal microscope (Carl Zeiss Microscopy, Oberkochen, Germany) for the experiments performed in Oslo. For the experiment executed in Boston, a Leica TCS-SP2 Upright Confocal Laser-Sanning Microscope was used. The number of live and dead cells (green and red fluorescence, respectively) was counted in five fields per sample at a magnification of 250x by two independent investigators. The percentage of viable cells per culture was calculated as live cells/(live cells + dead cells) × 100 (Table A in S1 Supplementary Data File). Three-week HLEC cultures (n = 2) exposed to methanol for 1 hour were used as positive controls for dead cells.

### Tissue Preparation

Non-stored and stored cultured HLEC were fixed in neutral buffered 4% formaldehyde and embedded in paraffin. Serial sections of 3.5 *μ*m were mounted on SuperFrost®Plus slides (Menzel-Glaser, Braunschweig, Germany), stained with hematoxylin and eosin (H&E) for histochemical and morphological analysis or incubated with antibodies for immunohistochemical analysis (Table 1). The immunohistochemical analysis was performed using Benchmark XT Antibody diluent (251–018) and Detection Kit Ventana ultraView Universal DAB (760–500), an automated immunostaining system based on the ABC avidin-biotin-peroxidase method, with negative and positive controls (Ventana Medical Systems Inc, Tucson, AZ, USA). Slides were incubated at 37°C overnight.

**Table 1:** Antibodies used in the study.

Antigen	Dilution	Clone	Company
ΔNp63α	1:200		Primm, Milano, Italy
p63	1:25	p63 protein mouse monoclonal clone 4A4. Code No M72747	DAKO Cytomation Norden A/S, Glostrup, Denmark
Bmi-1	1:20	Bmi-1 antibody, Rabbit, polyclonal, ab97729	Abcam, Cambridge, MA, USA
C/EBPδ	1:100	C/EBPδ antibody, Rabbit, polyclonal, ab65081	Abcam
ABCG2	1:20	ABCG2 protein, Mouse, monoclonal clone bxp-21.	Sigma Aldrich, St Louis, MO, USA
Cx-43	1:500	Rabbit, polyclonal, product no C6219	Sigma Aldrich
K19	1:200	CK19, Mouse, monoclonal RCK108.	DAKO
K3	1:500	K3, Clon AE5 Mouse anti-cytokeratin	ImmuQuest, Cleveland, UK
PCNA	1:3500	PCNA, Mouse monoclonal, M879	DAKO
Caspase-3	1:200	Cleaved Caspase-3, Asp175, 5A1E, Rabbit monoclonal antibody	Cell Signaling, Danvers, MA, USA

### Histological analysis

The H&E sections (n = 33) were photographed at magnification of 40x with a light microscope camera. The thickness of the cultured HLEC sheets (HAM not included) was measured on four pre-defined positions for both stored and non-stored cultures, using a digital imaging software (cell^p from Olympus Oslo, Norway). The predefined positions were 250 *μ*m, 500 *μ*m, 750 *μ*m, and 1000 *μ*m from the edge of the limbal explant (Table B in S1 Supplementary Data File). In addition, the thickness of the HAM on the same four pre-defined positions were measured for the non-stored cultures for all three experiments (Table C in S1 Supplementary Data File). The average central thicknesses per sections were calculated based on these four points of measurement.

### Immunohistochemical Analysis

Two independent investigators counted negative and positive cells through a light microscope at 400x magnification based on a pre-set list of criteria. The expression of the various markers per culture was calculated as follows: (the number of positive cells/total number of cells) × 100. The results from the two investigators were averaged (Table D, E, F, G, H, I, J, K, L and M in S1 Supplementary Data File). In addition, observer agreement was assessed for each marker as the mean difference between observer A and B with a 95% confidence interval (Table 2).

**Table 2:** Observer agreement.

Marker	Mean Difference Between Observers (%)	95% Confidence Interval for Mean Difference between Observers (%)
ΔNp63α	0.5	-0.7 to 1.8
p63	2.1	0.3 to 3.9
Bmi-1	0	-0.5 to 0.5
C/EBPδ	1.7	0.2 to 3.3
ABCG2	-1.4	-4.5 to 1.7
Cx-43	-2.5	-4.5 to-0.5
K19	2.2	-0.2 to 4.6
K3	-1.3	-4.5 to 1.9
PCNA	7	4.1 to 9.8
Caspase-3	0.3	-0.1 to 0.6

To explore possible regional variations in phenotype within the cultured epithelia (explants not included), we assessed the expression of all the markers in the basal and supra-basal epithelial layers separately after a grading system [29]. Cells that grew under or covered the explants were not included in the analysis. Each marker was graded as 0 (undetectable), + (detectable positivity in < ¼ of the cells), + + (detectable positivity in ¼—½ of the cells), + + + (detectable positivity in ½—¾ of the cells), and + + + + (detectable positivity in > ¾ of the cells) ().

### Statistical analyses

One-way analysis of variance (ANOVA) was used to compare the four different storage groups to a non-stored control group, with respect to cell viability and immunohistochemical markers. To correlate for multiple comparisons, Tukey’s post hoc test was used if equal variances were verified by Levene’s test. If equal variances were not verified by Levene’s test, the Dunnett’s T3 post hoc test was applied.

Pearson’s correlation test was used to examine co-variations between the thickness of the cultures and immunohistochemical markers, apart from one marker (Caspase-3) where Spearman’s Rank correlation test was used. The results from the five experimental groups are presented as mean ± standard error of the mean (SEM). A significance level of 5% was used throughout the study (SPSS version 21.0; SPSS Inc., Chicago. IL).

## Results

### Cell Viability for Non-stored and Stored Epithelia

**Fig 2:**
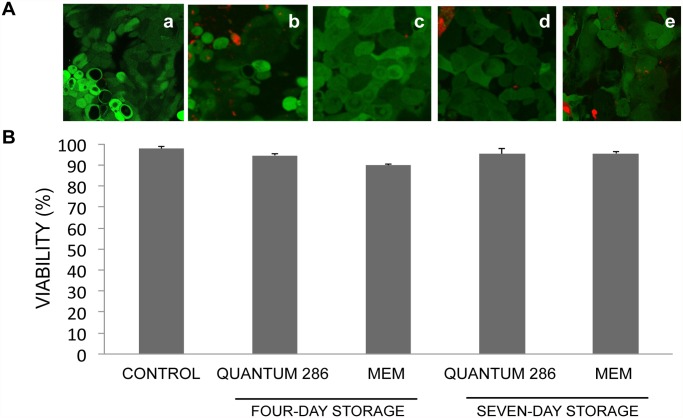
Mean Cell Viability of Non-Stored and Stored Cultured Limbal Epithelial Cells. A) Laser confocal micrographs following viability staining of cultured HLEC a) not subjected to storage, b) stored for 4 days in Quantum 286, c) stored for 4 days in MEM, d) stored for 7 days in Quantum 286 and e) stored for 7 days in MEM. Live cells are CAM+ (green), whereas dead cells are EH-1+ (red). Original magnification x250. B) Bar chart illustrating the percentage of viable cells in non-stored and stored cultured HLEC sheets for 4 and 7 days at 23°C in Quantum 286 or MEM. Error bars: Standard error of the mean.

The cell viability for the non-stored control group was 98 ± 1% (Fig. 2, Table A in S1 Supplementary Data File). After storage in MEM and Quantum 286 for 4 and 7 days, the cell viability remained high without any significant changes compared to the non-stored control group (Fig. 2, Table A in S1 Supplementary Data File). The percentage of viable cells per culture varied from a minimum of 83% (a culture stored in Quantum 286 for 7 days) to a maximum of 100% (a culture stored in MEM for 4 days) (Table A in S1 Supplementary Data File).

### Variations in Histological Appearance and Thickness of HAM and HLEC Sheets

**Fig 3:**
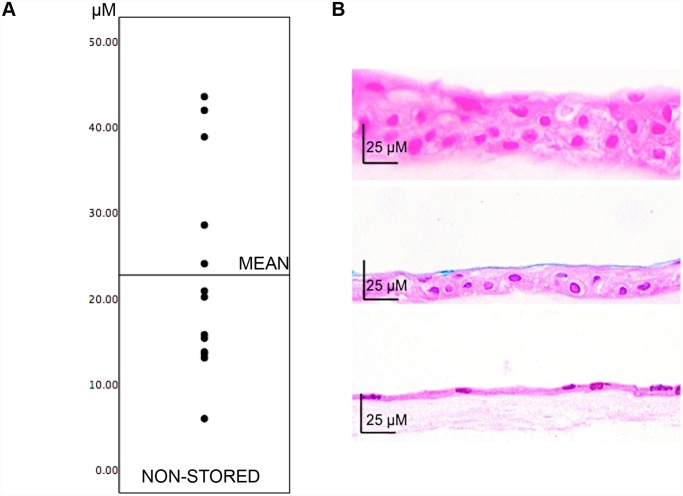
Variations in Epithelial Sheet Thickness Between Cultures. A) Central thickness of all non-stored epithelial sheets plotted in ascending order (amniotic membranes not included in the measurements). B) Hematoxylin-Eosin sections of epithelial sheets with a) maximum thickness. b) average thickness and c) minimum thickness. Original magnification x400.

For the non-stored cultured epithelia, the central epithelial sheet thickness (250–1000 *μ*m from the explant) varied between 5.9 *μ*m and 43.5 *μ*m, with an average of 23 ± 3 *μ*m (Fig. 3A, Table B in S1 Supplementary Data File). In the culture with an average thickness of only 5.9 *μ*m, the epithelium was arranged in a monolayer. For all other thicknesses, the epithelia contained two or more layers, with cuboid wing epithelial cells in the basal and intermediate layers and squamous epithelial cells in the superficial layer. The non-stored epithelia were well attached to the amniotic membranes, and there were no signs of shedding of cells (Fig. 3B).

Central HAM thickness (250–1000 *μ*m from the explant) varied between 13 *μ*m and 304 *μ*m, with a mean of 83 ± 24.7 *μ*m. The HAMs consisted of a basal membrane with underlying stroma containing organized fibrils and scattered stromal cells (Fig. 3B, Table C in S1 Supplementary Data File).

**Fig 4:**
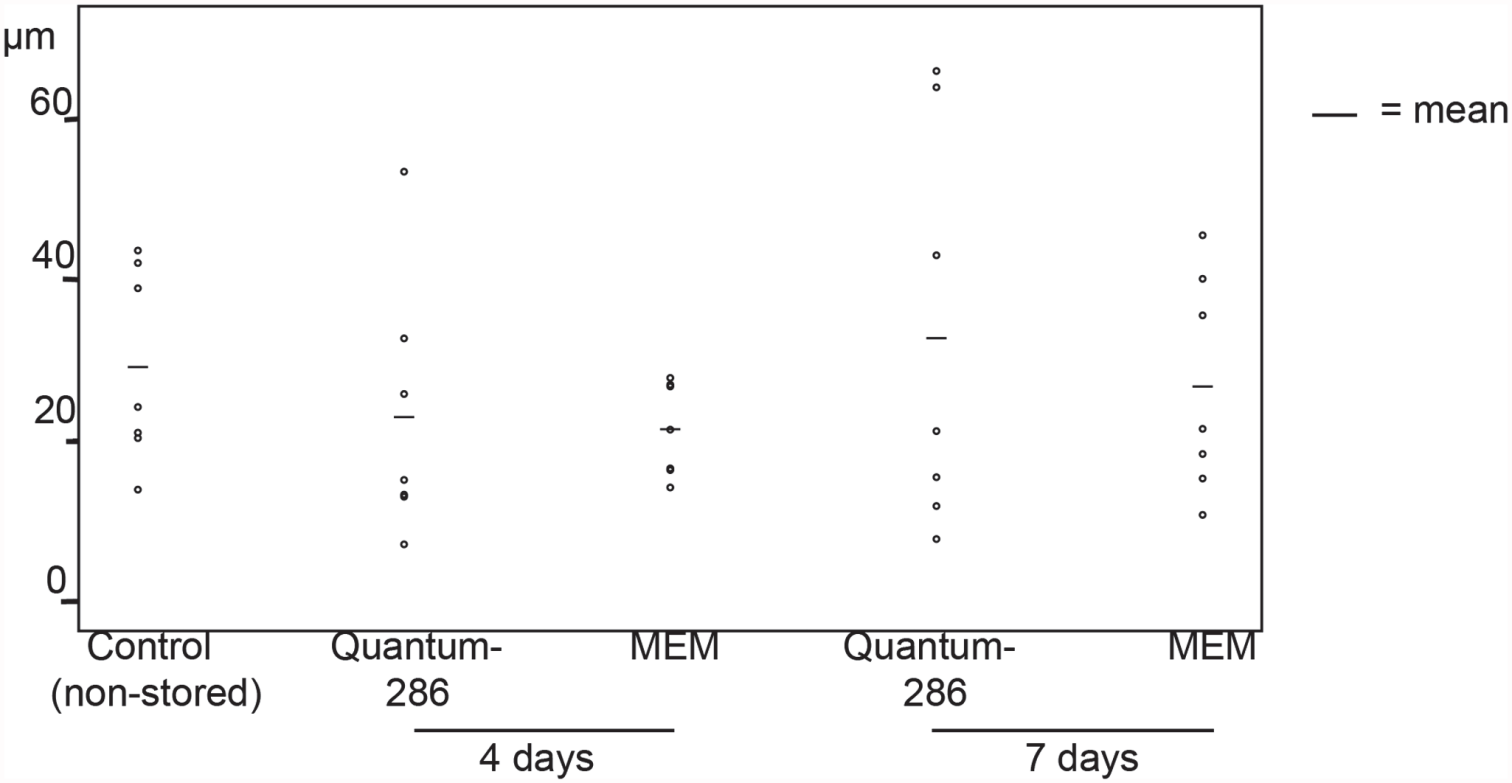
Mean Central Thickness of Non-Stored and Stored Cultured Limbal Epithelial Sheets. Scatterplot illustrating average central thickness (*μ*m) of cultured human limbal epithelial cell sheets stored for 4 and 7 days in Quantum 286 or MEM, compared to a non-stored control group. Horizontal bars represent mean thickness per group.

Central thickness of non-stored versus stored cultured HLEC sheets also varied (experiment 1 and 2, a total of 35 cultures, Fig. 4, Table B in S1 Supplementary Data File). However, there were no lost cultured HLEC sheets after storage, demonstrated by the H&E sections. Occasionally, focal detachment of cells from HAM was seen, but not more in stored than non-stored cultures. More shedding of cells from the superficial layers was observed in stored cultures compared to non-stored. In summary, even though sporadic shedding of cells occurred, no substantial loss of cells was seen for stored HLEC sheets.

### Expression of Markers for Immaturity, Differentiation, Proliferation and Apoptosis

Possible variations in differentiation, proliferation, and apoptosis of cells between non-stored and stored cultured HLEC sheets were assessed through extensive immunhistochemical analyses.

**Fig 5:**
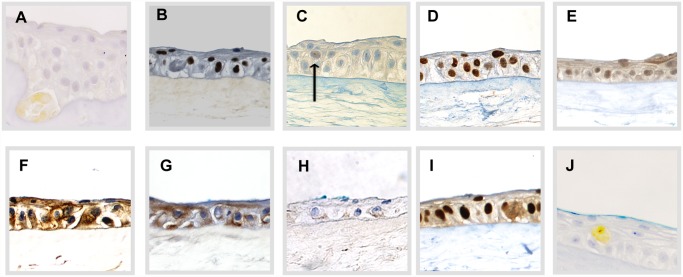
Staining Pattern of Immunohistochemical Markers Used in the Study. Staining Pattern of Immunohistochemical Markers from representative areas of Non-Stored Cultured Limbal Epithelial Cell Sheets. The pictures show nuclear markers A) ΔNP63α, B) pan-p63, C) Bmi-1, and D) C/EBP∂; membrane markers E) ABCG2, and F) Cx-43; keratin markers G) K19, and H) K3, proliferating cell marker I) PCNA, and finally, apoptosis marker J) Caspase-3. Original magnification 400x. Arrow in C) marks positively stained cell.

**Fig 6:**
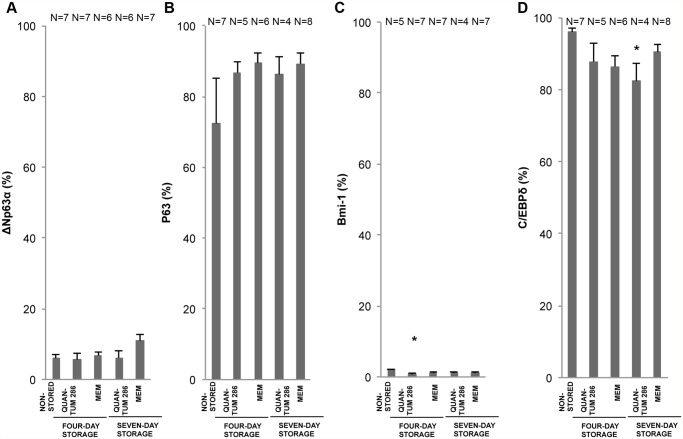
Holoclone-Associated Marker Expression for Non-Stored and Stored Cultured Limbal Epithelial Cells. Bar chart of mean percentage of positively stained cells for 4 different storage conditions compared to a non-stored control group of cultured human limbal epithelial cells. The markers are A) ΔNP63α, B) pan-p63 (4A4 antibody), C) Bmi-1, and D) C/EBP∂. Error bars: Standard Error of the Mean. * *P*<0.05 compared to the non-stored control group. N = Number of cultures.

The holoclone-associated ΔNα isotype of p63 [23,24] was compared with pan-p63 [30] and the holoclone-associated markers CCAAT/enhancer binding protein delta (C/EBP∂) and Bmi-1 [24]. The expression of ΔNp63α and pan-p63 was found in the basal layer of the epithelia, and pan-p63 was also expressed in the supra-basal layers. The ΔNp63α staining appeared yellow. ΔNp63α- expression appeared sometimes as solitaire positive cells and sometimes as clusters of basal or intermediate, positively stained cells. Occasionally, ΔNp63α-positive cells were found in the supra-basal layers (Table 3, Fig. 5A and B). ΔNp63α was positive in 4.6 ± 1% of the cells in the non-stored HLEC sheets (Fig. 6A, Table D in S1 Supplementary Data File), while the pan-p63 positivity was considerably higher (77 ± 6%) (Fig. 6B, Table E in S1 Supplementary Data File). A strong ΔNp63α positivity of more than 3%, associated with a high success rate after transplantation [21,23], was achieved in about ⅔ of the HLEC cultures from explants (9 of 13 cultures) (Table D in S1 Supplementary Data File). No significant changes of pan-p63 and ΔNp63α expression were observed for any of the storage conditions compared to the non-stored group (Fig. 6A and B).

**Table 3:** Expression of immunohistochemical markers in basal versus suprabasal layers.

		Non-stored	Quantum 286 4 days	MEM 4 days	Quantum 286 7 days	MEM 7 days
ΔNP63α	sb ^1^	(+)^2^	(+)	(+)	(+)	(+)
	b ^3^	+^4^	+	+	+	+
p63	sb	+++^5^	+++	+++	+++	+++
	b	++++^6^	++++	++++	++++	++++
Bmi-1	sb	+	0^7^	0	0	0
	b	+	+	+	+	+
C/EBP∂	sb	+++(+)^8^	+++	+++	+++	+++
	b	++++	++++	++++	++++	++++
ABCG2	sb	++++	++++	++++	+++	++++
	b	++++	++++	++++	+++(+)	++++
Cx43	sb	++++	++++	++++	++++	++++
	b	++++	++++	++++	++++	++++
K19	sb	+++(+)	+++	+++(+)	+++(+)	+++(+)
	b	++++	++++	++++	++++	++++
K3	sb	+(+)^9^	+(+)	++^10^	++(+)	++(+)
	b	+	+	+	++	++
PCNA	sb	+++(+)	+++	+++(+)	+++	+++
	b	++++	++++	++++	++++	++++
Caspase-3	sb	(+)	(+)	(+)	+	(+)
	b	(+)	(+)	(+)	+	(+)

^1^ sb = supra-basal

^2^ (+) = mostly detectable in < ¼ of cells, not detectable in some sections

^3^ b = basal

^4^ + = detectable in < ¼ of cells

^5^ +++ = detectable in ½—¾ of cells

^6^ ++++ = detectable in > ¾ of cells

^7^ 0 = not detectable

^8^ +++(+) = mostly detectable in > ¾ of cells, detectable in ½—¾ of cells for some sections

^9^ +(+) = mostly detectable in ¼—½ of cells, detectable in < ¼ of cells for some sections

^10^ ++ = detectable in ¼—½ of cells.

Bmi-1-expression resembled the relatively low ΔNp63α expression, while C/EBP∂ expression was far more abundant, corresponding to pan-p63. Bmi-1 was mostly expressed in the basal layer of the epithelia, with an average positivity of 3 ± 1% for the non-stored group. Occasionally, Bmi-1 expression was seen in solitaire cells in supra-basal layers (Table 3, Fig. 5C). Bmi-1 expression was significantly reduced from 2.0 ± 0.3% to 0.7 ± 0.3% after storage in Quantum 286 for 4 days (P = 0.02) (Fig. 6C, Table F in S1 Supplementary Data File). C/EBP∂ was positive in 95 ± 1% of the non-stored cells, with expression in both basal and supra-basal layers (Table 3, Fig. 5D, Table G in S1 Supplementary Data File). C/EBP∂ declined to 82 ± 5% after 7- day Quantum 286-storage (P = 0.04) (Fig. 6D, Table G in S1 Supplementary Data File). There were no significant changes in expression of Bmi-1 and C/EBP∂ after MEM-storage (Fig. 6C and D).

**Fig 7:**
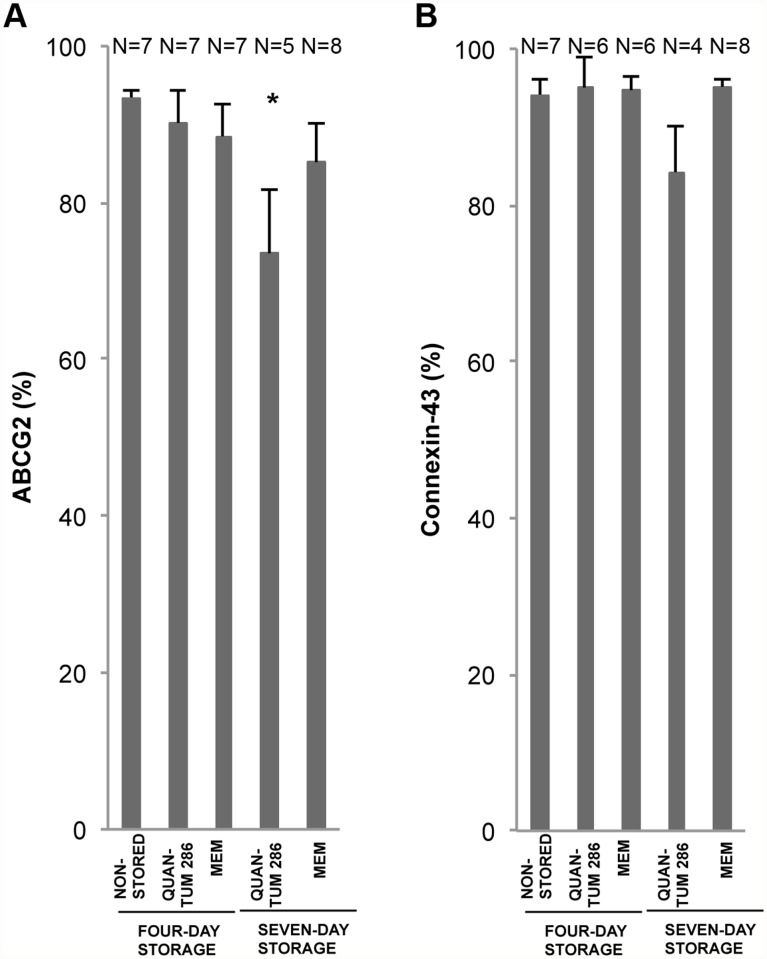
Membrane Marker Expression for Non-Stored and Stored Cultured Limbal Epithelial Cells. Bar chart of mean percentage of positively stained cells for 4 different storage conditions compared to a non-stored control group of cultured human limbal epithelial cells. The markers are A) ABCG2, and B) Cx-43. Error bars: Standard Error of the Mean. * *P*<0.05 compared to the non-stored control group. N = Number of cultures.

The two membrane proteins ABCG2, a marker of immature cells [31], and connexin-43, generally viewed as differentiation marker [32], were compared. ABCG2 was highly expressed in all layers of the non-stored epithelial sheets (Table 3, Fig. 5E), with an average positivity of 91 ± 5% (Table H in S1 Supplementary Data File). The expression decreased to 74 ± 8% after Quantum 286-storage for 7 days (P = 0.04) but was unchanged for the other storage conditions (Fig. 7A, Table H in S1 Supplementary Data File). Surprisingly, the putative differentiation marker Connexin-43 was high, at a level of 94 ± 1% throughout the epithelia (Table 3, Fig. 5F, Table I in S1 Supplementary Data File), with no significant changes after storage (Fig. 7B, Table I in S1 Supplementary Data File).

**Fig 8:**
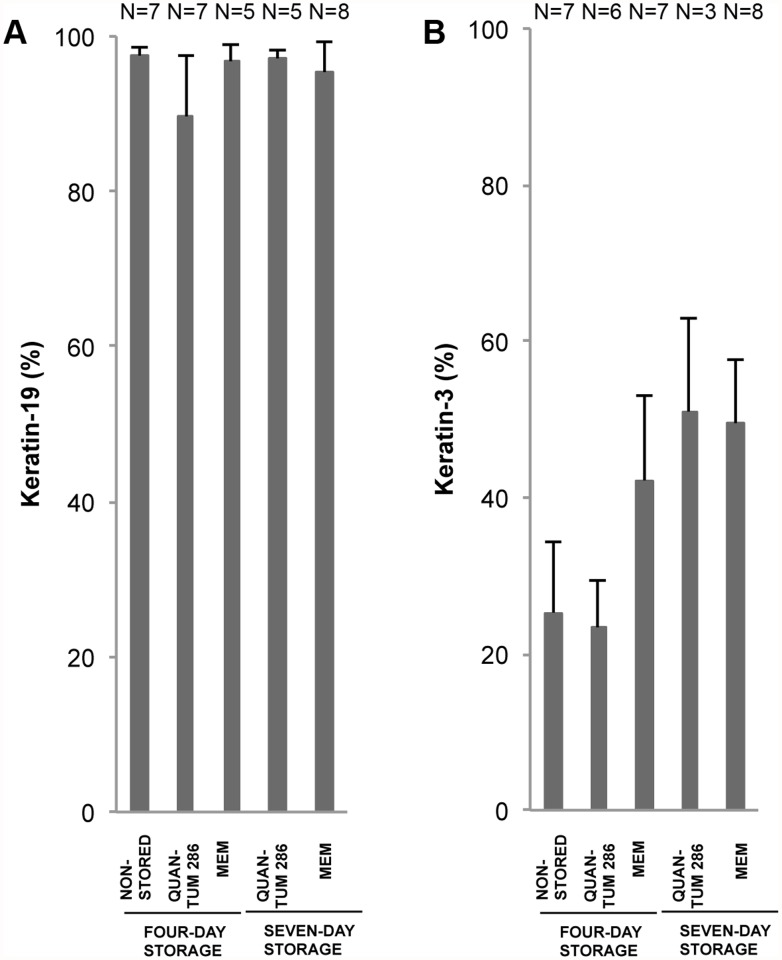
Keratin Marker Expression for Non-Stored and Stored Cultured Limbal Epithelial Cells. Bar chart of mean percentage of positively stained cells for 4 different storage conditions compared to a non-stored control group of cultured human limbal epithelial cells. The markers are A) K19, and B) K3. Error bars: Standard Error of the Mean. N = Number of cultures.

We looked at two keratins; keratin-19 (K19), suggested to mark immature cells [31], and keratin-3 (K3), known to mark terminally differentiated corneal cells [2]. K19-staining occurred in both basal and supra-basal layers (Table 3, Fig. 5G). 89 ± 6% of non-stored cells stained positively for K19, with no significant changes after storage (Fig. 8A, Table J in S1 Supplementary Data File). K3 was weakly expressed in both basal and supra-basal layers (mean 28 ± 5%), with no significant changes after storage for any of the storage conditions (Table 3, Fig. 5H and 8B, Table K in S1 Supplementary Data File).

**Fig 9:**
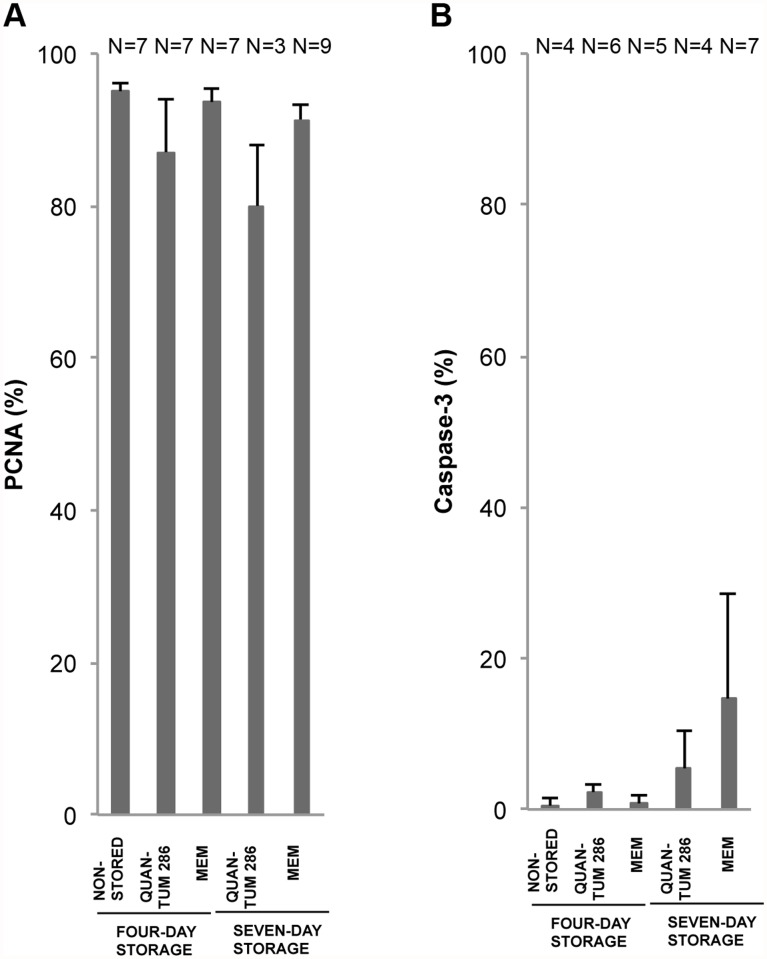
Proliferation and Apoptosis Marker Expression for Non-Stored and Stored Cultured Limbal Epithelial Cells. Bar chart of mean percentage of positively stained cells for 4 different storage conditions compared to a non-stored control group of cultured human limbal epithelial cells. The markers are A) PCNA, and B) Caspase-3. Error bars: Standard Error of the Mean. N = Number of cultures.

Finally, the number of proliferating versus apoptotic cells was assessed by staining with Proliferative Cell Nuclear Antigen (PCNA) [33] and Caspase-3 [34], respectively. The expression of PCNA in non-stored HLEC sheets was high in both basal and supra-basal layers (Table 3, Fig. 5I), with a mean of 94 ± 2% (Fig. 9A, Table L in S1 Supplementary Data File). The Caspase-3 expression was low with an average of 0.5 ± 0.5% before storage (Table 3, Fig. 5J and 9B, Table M in S1 Supplementary Data File). The Caspase-3 staining appeared yellow. No significant changes in PCNA and Caspase-3 expression were observed after storage for any of the storage conditions (Fig. 9A and B, Table L and M in S1 Supplementary Data File).

In summary, an overall pattern of a predominantly immature phenotype with highly proliferative epithelial cells and low apoptotic values was maintained after storage. There were no significant changes in expression for any of the immunohistochemical markers after storage in MEM; however, for storage in Quantum 286, three of the immature phenotype markers (Bmi-1, C/EBP∂, ABCG2) showed a reduced expression compared to the non-stored control group. Hence, MEM-storage tended to preserve an immature phenotype better than storage in Quantum 286.

### Correlations between Epithelial and HAM Thickness and Phenotype

**Fig 10:**
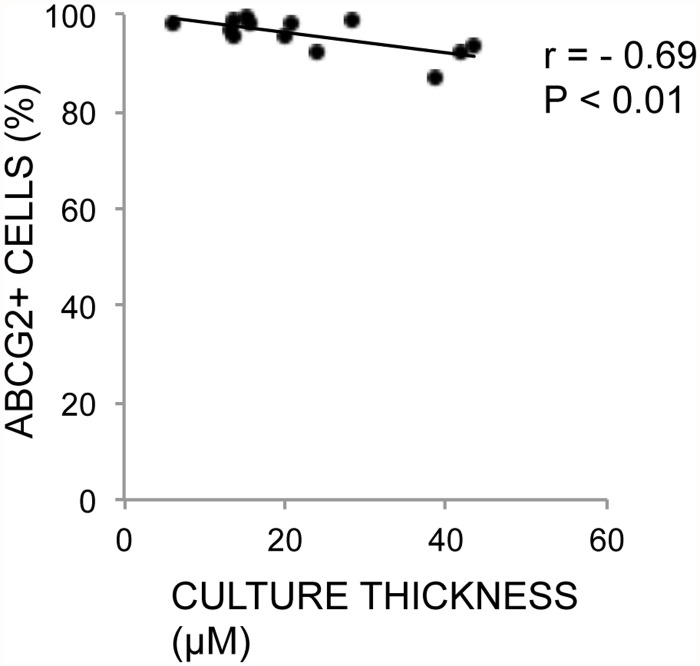
Correlation Between ABCG2 Expression and Thickness of Non-Stored Cultures. Scatterplot with trend line of average thickness per culture (x-axis) against percentage of ABCG2-positive cells per culture (y-axis) for 13 non-stored human limbal epithelial cultures. r = Pearson’s correlation coefficient. P = p-value for Pearson’s correlation test.

**Fig 11:**
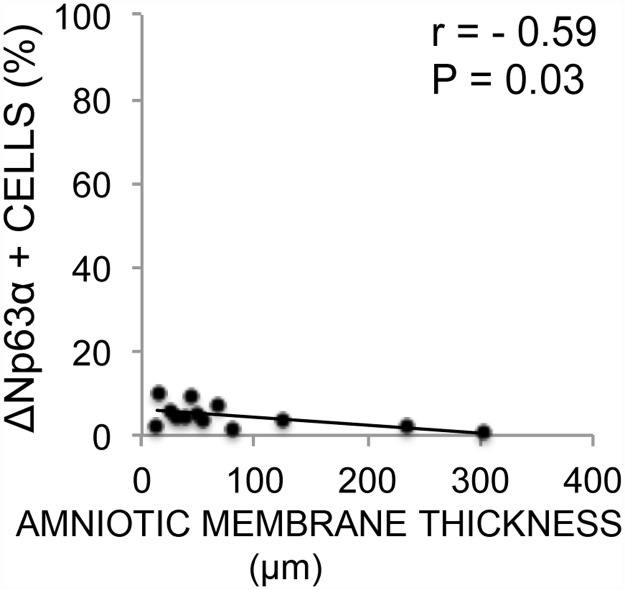
Correlation Between ΔNP63α Expression and Thickness of Amniotic Membranes. Scatterplot with trend line of average thickness of amniotic membrane substrate (x-axis) against percentage of ΔNP63α-positive cells per corresponding culture (y-axis) for 13 non-stored human limbal epithelial cultures. r = Pearson’s correlation coefficient. P = p-value for Pearson’s correlation test.

Expression of each of the immunohistochemical markers was correlated with the average epithelial thickness and average HAM thickness in their respective cultures (non-stored). A negative correlation between the cultured epithelial sheet thickness and ABCG2 was revealed (r = -0.69, P < 0.01) (Table 4, Fig. 10) as well as a negative correlation with HAM thickness and ΔNp63α expression (r = -0.59, P = 0.03) (Table 5, Fig. 11). Epithelial thickness and HAM thickness did not correlate (r = 0.15, P = 0.63). The remaining markers did not correlate significantly with thickness of either the cultured HLEC or the cultured HAM (Tables 4 and 5).

**Table 4:** Correlations between cultured epithelial thickness and immunohistochemical markers.

Marker	Mean expression / culture (%)	Correlation Coefficient	P-value (2-tailed)
ΔNP63α	4.6	-0.1	0.88
p63	77.3	0.4	0.2
Bmi-1	3.1	-0.1	0.78
C/EBP∂	95	-0.1	0.79
ABCG2	91.4	-0.69*	0.01*
Cx43	93.9	0.2	0.44
K19	88.6	0.5	0.14
K3	27.6	-0.1	0.64
PCNA	94.1	-0.1	0.85
Caspase-3	0.3	0.0	0.99

**Table 5:** Correlations between amniotic membrane thickness and immunohistochemical markers.

Marker	Mean expression / culture (%)	Correlation Coefficient	P-value (2-tailed)
ΔNP63α	4.6	-0.59*	0.03*
p63	77.3	0.29	0.33
Bmi-1	3.1	0.26	0.44
C/EBP∂	95	0.15	0.63
ABCG2	91.4	-0.07	0.82
Cx43	93.9	0.13	0.67
K19	88.6	0.01	0.98
K3	27.6	-0.10	0.75
PCNA	94.1	0.10	0.75
Caspase-3	0.3	-0.22	0.54

## Discussion

In the present study, the cultured HLEC sheets were stored for 4–7 days. Storage, in the present context, means that cells are kept in sealed containers with no adjustments of atmosphere. This is radically different from the 14 days of incubation in humidified 5% CO_2_ atmosphere prior to storage. During storage, the epithelial cells are not static, as one would expect with cryopreservation, even if the lowered temperature (from 37 to 23°C) most likely slows down cellular metabolism. The lack of medium change and non-physiologic temperature could cause cell death and detachment from the substrate. However, we found that the epithelial cell sheets were unchanged after storage with respect to cell viability, with a preserved, predominantly immature phenotype and no substantial loss of cells.

Cell viability was 98 ± 1% after 14 days of culture, with no significant changes for the groups with additional 4 and 7 days of serum-free storage, which is in line with our previous studies of storage of cultured HLEC using a serum-based storage medium [35,36]. Interestingly, by extending the culture period of HLEC from 15 to 28 days, Hayashi *et al*. noted a drop in cell viability from 93.2% to 64.1% [37]. Based on this, storage under the above mentioned conditions, rather than prolonged culture time, could protect against cell death.

Dead cell debris suspended in the medium is not accounted for, which could mean that a possible depletion in the absolute number of live cells in the stored versus non-stored epithelia is missed. To overcome this problem, only cell viability measurements from the basal layer of the epithelia were included. Dead basal layer cells are less likely to detach since they are enclosed between adjacent cell layers and the amniotic membrane. In addition, the study includes morphological analyses such as microscopy of H&E sections and thickness measurements, demonstrating no substantial loss of cells.

The storage conditions with 23°C, MEM or Quantum 286, and 4–7 days storage protected against cell death, loss of ultrastructure, and differentiation of cells. Firstly, the choice of temperature is important for successful preservation of cells. Raeder *et al*. demonstrated that storage of cultured HLEC at 23°C was superior to both 4 and 31°C in maintaining their morphology [35]. Reduced epithelial thickness has also been registered following storage of whole corneas at 34°C [38] and 37°C [39,40]. Therefore, the preservation of epithelial morphology may be partially explained by our choice of temperature.

In addition, the choice of storage media in the present study is likely to play a role in the preservation of the epithelia. Storage in MEM, a simple storage medium, preserved an immature phenotype better than the more complex Quantum 286. Simple media may reduce the metabolism, and thereby potentially avoid differentiation during storage to a higher extent than media with added growth factors, such as Quantum 286. This finding is in agreement with another study demonstrating increased differentiation of cultured HLEC over time using a complex culture medium [41]. Future studies correlating metabolic parameters in various storage media with the phenotype of cultured HLEC are warranted.

Since the cells were initially cultured in a medium with added 10% FBS, one could argue that a certain amount of serum would be transferred from the culture medium to the storage media, for example contained in the human amniotic membrane, so that the storage would not be under complete serum-free conditions. Serum amounts of even less than 0.5% can have a sustainable effect on cells [42]. However, the unintended serum concentration in the storage containers would be markedly lower than this level because 1) effort was taken to remove as much culture medium as possible, and 2) each epithelial sheet was submerged in storage medium about 10 times the volume of the culture wells. Therefore, although a possible serum effect on the stored cells cannot be totally excluded, it is most likely negligible as a cell-preserving factor.

Nevertheless, the minimal serum transferral from culture to storage containers carries a potential risk of animal infectious agents being passed on to the end product. Therefore, for clinical use, a protocol free of animal components both for culture and storage should be followed in order to eliminate the risk of transmission of animal-borne diseases.

Also, other unknown factors could add to the preservation of the epithelial sheets, such as the choice of HAM as a substrate, the explant remaining on the culture all through the storage time, and the conditions the epithelial sheets were in before storage was started. Further studies are needed to investigate if serum-free/xeno-free storage can also be performed, for example, with epithelia cultured on artificial substrates from cell suspension.

Despite identical culture conditions, a considerable difference in central thickness of the non-stored, cultured limbal epithelial sheets was noted, ranging from monolayer to stratified, multi-layered epithelium. These findings are in agreement with another study from our group [43].

Mechanical properties like thickness of the substrate have recently become highly relevant, with Chen *et al*.’*s* work in 2012 demonstrating a higher differentiation (by K3 expression) of limbal cells cultured on HAM measured as soft (by shear rheology) and thick (mean thickness 115.6 ± 20.7*μ*m) compared to differentiation level of cells cultured on stiffer and r HAM. A similar association between stiffness and limbal epithelial differentiation is found for an artificial substrate [44]. Surprisingly, we found a significant negative correlation between HAM thickness and expression of the putative stem cell marker ΔNp63α. Storage studies that include testing of HAM stiffness and other mechanical properties are needed to investigate this result further.

A negative correlation with the putative stem cell marker ABCG2 and epithelial thickness was found in our study. Air-lifting is a culture technique where the medium level in the culture wells is reduced in order to promote stratification and strengthening of ultrastructure. A possible loss of ABCG2-positive putative stem cells with thickness and stratification, like the correlation observed in the present study suggests, would be an argument against the air-lifting technique in production of LEC transplants. This assumption is supported by a study showing differentiation and gradual loss of stem cells following air-lifting [45]. However, our findings are ambiguous, since apart from ABCG2, the expression of all other stem cell and differentiation markers was found to be unrelated to epithelial cell thickness. Most importantly, the expression of ΔNp63α, which is associated with a good long-term prognosis after transplantation [21], did not correlate with thickness. This again suggests that thin grafts may contain enough stem cells to be suitable for transplantation.

A thin epithelium may contain enough stem cells, but a confluent and well-stratified epithelium may be more resilient to handling and mechanical stress, and this would be preferred for transplants. Hence, a thin epithelial graft should be large enough to cover the damaged cornea and the limbal region and, in combination with the substrate, have sufficient mechanical strength to endure the surgery and the postoperative period [41,46,47].

The immunohistochemical markers used in the study demonstrated a pattern of predominantly immature cells for both non-stored and stored HLEC sheets. Barbaro *et al*. showed that co-expression of C/EBPδ, Bmi-1 and ΔNp63α transcription factors identify mitotically quiescent limbal stem cells, which generate holoclones in culture [24]. C/EBPδ-staining was restricted to a subpopulation of ΔNp63α-positive cells [24]. In contrast, we noted a high expression of C/EBPδ and a low expression of Bmi-1 and ΔNp63α in cultured HLEC. Barbaro *et al*. investigated the relationship between C/EBPδ, Bmi-1 and ΔNp63α in cultures from holoclones isolated from limbal epithelial cells [24], while our study analyzed unselected epithelial cells cultured from limbal explants. The difference in C/EBPδ expression between the studies may indicate a less specific staining pattern of C/EBPδ when exposed to cells of various clonal origins, rather than solely holoclones. A recent study by Borderie *et al*. supports this notion [41]. Bmi-1, however, with its very low expression and basal location in our study, could be a better candidate as a stem cell marker in combination with ΔNp63α.

In our study, we used the 4A4 antibody to detect pan-p63. High expression of p63 in cultured HLEC is in accordance with several previous studies [35,36]. The low expression of the ΔN-α isotype of p63, combined with its predominantly basal location, further support the notion of ΔNp63α as a stem cell marker. P63 has been found to mark cells in a proliferative state [48], which is supported by our high expression of PCNA.

The transmembrane transporter protein ABCG2 was highly expressed in both basal and supra-basal layers. ABCG2 has been suggested a putative stem cell marker from a wide variety of tissues [49,50]. However, in cultured limbal tissue, Dua *et al*. observed ABCG2-staining in the limbal region that was too high to be accounted for by stem cells alone, and suggested that ABCG2-positively stained cells could represent both stem cells and cells within their immediate progeny [31]. Our study confirms the conclusion by Dua *et al*.

A high expression of K19, typically found in the basal layer of the limbal region [51] and a low expression of the corneal differentiation marker K3 [2], further contribute to the finding of a generally immature phenotype of cultured HLEC. The expression of K3 in our study is lower than in several other studies, including one by Kim *et al*. where 62% of the cells expressed K3 [35]. This can be explained by our use of intact amniotic membrane, which is known to support an undifferentiated phenotype [52–54]. However, our Cx43 expression was higher than the study by Kim *et al*. Like K3, Cx43 is generally considered a marker of differentiation [2,55], even though it has also been proposed to mark transient amplifying cells in the vicinity of limbal stem cells, and hence denote limbal stem cells [32]. In contrast to K3, there are also reports demonstrating the role of Cx43 in maintaining stemness [56,57], which could be a possible explanation to the high expression of Cx43 found in the present study.

Although semi-quantitative evaluation [35] of immunostaining is more common than elaborate quantitative methods [41,58], we performed both to enable calculation of inter-observer agreement, while maintaining data on regional expression. Western blot, in contrast, requires significantly more protein and does not allow information about co-localization in cells nor in the tissue.

In the present study, a strong ΔNp63α positivity of more than 3%, associated with a high success rate after transplantation [21,23], was achieved in about ⅔ of the HLEC cultures from explants. The explants in the present study are harvested from necro-donors and cultures started up until three weeks post mortem. Further studies are warranted to demonstrate if the numbers are repeatable also with autologous tissue, living related donors, and necro-donors with shorter post-mortem time. However, if the ΔNp63α expression found in our study is representative for explant culture, it would indicate that almost one third of the cultured HLEC sheets from limbal explants are likely to fail after transplantation [21] unless grafts with a ΔNp63α expression below 3% are identified and excluded prior to surgery. The level of ΔNp63α expression found in our study is in line with the overall success rate of transplantation of cultured HLEC (70% for allografts and 75% for autografts) [4]. A storage interval of a few days after the culture time before transplantation would allow sufficient time to test the cultured LEC sheets for ΔNp63α expression and other parameters to ensure good quality of the grafts. The present study shows that this can be done, even in a storage medium without both serum and xenobiotics.

## Conclusion

After culture of HLEC sheets from explants on HAM, the cultured limbal epithelial cells can be stored at 23°C in both serum-free and xenobiotic-free media for 4 and 7 days, with sustained cell viability, ultrastructure, and maintained expression of immature phenotype markers, including ΔNp63α. Hence, our study contributes to establishing a serum- and xenobiotic-free chain for limbal stem cell therapy.

## Supporting Information

S1 Supplementary Data File Cell Viability, Epithelial and Human Amniotic Membane Thickness and Immunohistochemistry. S1 Supplementary Data File presents data underlying the analyses in the manuscript. The file consists of 13 sheets containing one table per sheet (Table A, B, C, D, E, F, G, H, I, J, K, L and M), referred to in the text as “Table A in S1 Supplementary Data File” etc. Table A”Cell Viability” contains the percentage of viable cells per sample. Table B “Epithelial Thickness” contains the thickness of the epithelial sheets (amniotic membrane not included) in *μ*m measured on four pre-set spots; 250, 500, 750 and 1000 *μ*m from the explant. Table C “Human Aminotic Membrane Thickness” contains the thickness of the amniotic membrane in *μ*m measured on four pre-set spots; 250, 500, 750 and 1000 *μ*m from the explant of each sample. The remaining tables contain supplementary data of the expression of the 11 different immunohistochemical markers examined by two investigators from one section per culture. Each table is named after the marker presented (table D = ΔNp63α, table E = P63, table F = Bmi-1, table G = C/EBP∂, table H = ABCG2, table I = Connexin-43, table J = Keratin-19, table K = Keratin-3, table L = PCNA, table M = Caspase-3).
(XLSX)
